# Overlapping and Distinct Features of Cardiac Pathology in Inherited Human and Murine Ether Lipid Deficiency

**DOI:** 10.3390/ijms24031884

**Published:** 2023-01-18

**Authors:** Fabian Dorninger, Attila Kiss, Peter Rothauer, Alexander Stiglbauer-Tscholakoff, Stefan Kummer, Wedad Fallatah, Mireia Perera-Gonzalez, Ouafa Hamza, Theresa König, Michael B. Bober, Tiscar Cavallé-Garrido, Nancy E. Braverman, Sonja Forss-Petter, Christian Pifl, Jan Bauer, Reginald E. Bittner, Thomas H. Helbich, Bruno K. Podesser, Hannes Todt, Johannes Berger

**Affiliations:** 1 Department of Pathobiology of the Nervous System, Center for Brain Research, Medical University of Vienna, Spitalgasse 4, 1090 Vienna, Austria; 2 Center for Biomedical Research, Medical University of Vienna, Währinger Gürtel 18-20, 1090 Vienna, Austria; 3 Department of Neurophysiology and Neuropharmacology, Center for Physiology and Pharmacology, Medical University of Vienna, Währingerstrasse 13a, 1090 Vienna, Austria; 4 Department of Biomedical Imaging and Image-Guided Therapy, Division of Molecular and Structural Preclinical Imaging, Medical University of Vienna, Währinger Gürtel 18-20, 1090 Vienna, Austria; 5 Neuromuscular Research Department, Center for Anatomy and Cell Biology, Medical University of Vienna, Währinger Straße 13, 1090 Vienna, Austria; 6 Department of Genetic Medicine, King AbdulAziz University, Jeddah 21589, Saudi Arabia; 7 Department of Human Genetics and Pediatrics, Montreal Children’s Hospital, McGill University, 1001 Décarie Blvd, Montreal, QC H4A 3J1, Canada; 8 Skeletal Dysplasia Program, Nemours Children’s Hospital, 1600 Rockland Road, Wilmington, DE 19803, USA; 9 Department of Pediatrics, Division of Cardiology, Montreal Children’s Hospital, McGill University, 1001 Décarie Blvd, Montreal, QC H4A 3J1, Canada; 10 Department of Molecular Neurosciences, Center for Brain Research, Medical University of Vienna, Spitalgasse 4, 1090 Vienna, Austria; 11 Department of Neuroimmunology, Center for Brain Research, Medical University of Vienna, Spitalgasse 4, 1090 Vienna, Austria

**Keywords:** plasmalogen, ether lipid, left ventricular hypertrophy, cardiac disease, peroxisome, rhizomelic chondrodysplasia punctata

## Abstract

Inherited deficiency in ether lipids, a subgroup of glycerophospholipids with unique biochemical and biophysical properties, evokes severe symptoms in humans resulting in a multi-organ syndrome. Mouse models with defects in ether lipid biosynthesis have widely been used to understand the pathophysiology of human disease and to study the roles of ether lipids in various cell types and tissues. However, little is known about the function of these lipids in cardiac tissue. Previous studies included case reports of cardiac defects in ether-lipid-deficient patients, but a systematic analysis of the impact of ether lipid deficiency on the mammalian heart is still missing. Here, we utilize a mouse model of complete ether lipid deficiency (*Gnpat* KO) to accomplish this task. Similar to a subgroup of human patients with rhizomelic chondrodysplasia punctata (RCDP), a fraction of *Gnpat* KO fetuses present with defects in ventricular septation, presumably evoked by a developmental delay. We did not detect any signs of cardiomyopathy but identified increased left ventricular end-systolic and end-diastolic pressure in middle-aged ether-lipid-deficient mice. By comprehensive electrocardiographic characterization, we consistently found reduced ventricular conduction velocity, as indicated by a prolonged QRS complex, as well as increased QRS and QT dispersion in the *Gnpat* KO group. Furthermore, a shift of the Wenckebach point to longer cycle lengths indicated depressed atrioventricular nodal function. To complement our findings in mice, we analyzed medical records and performed electrocardiography in ether-lipid-deficient human patients, which, in contrast to the murine phenotype, indicated a trend towards shortened QT intervals. Taken together, our findings demonstrate that the cardiac phenotype upon ether lipid deficiency is highly heterogeneous, and although the manifestations in the mouse model only partially match the abnormalities in human patients, the results add to our understanding of the physiological role of ether lipids and emphasize their importance for proper cardiac development and function.

## 1. Introduction

Human deficiency in the biosynthesis of ether lipids, a subgroup of glycerophospholipids, leads to multi-organ disease including severe dysfunction of the brain, respiratory, and visual system and massive malformations of the skeleton [1]. The corresponding inherited disease, rhizomelic chondrodysplasia punctata (RCDP), is a rare condition with an estimated incidence of 1:100,000. It can be caused by mutations in five different genes (*PEX7*, *GNPAT*, *AGPS*, *FAR1,* and *PEX5* (long isoform) causing RCDP types 1–5, respectively), all involved in different peroxisomal steps of the biosynthesis pathway [2,3,4,5,6]. How the lack of ether lipids impairs organ function on the molecular level is in most cases not established, probably due to the complex and manifold functions and properties that ether lipids confer to lipid membranes. Several subtypes of ether lipids exist. They all have in common the characteristic ether bond at the *sn*-1 position of the glycerol backbone and share the same biosynthesis pathway, which requires peroxisomes for the first crucial steps [7]. The most prominent ether lipids are plasmalogens, for which several molecular functions have been proposed, like signal transduction [8] and the generation of second messengers as well as antioxidative activity, which is controversially discussed in recent literature (see [9,10] for review). Most importantly, though, plasmalogens are major membrane structural constituents fine-tuning biophysical properties like membrane fluidity or curvature [11].

Cardiac tissue is rich in plasmalogens, particularly those with a choline headgroup as opposed to ethanolamine headgroups, which are more prominent in most other tissues [12]. A recent study suggested that cardiac choline plasmalogens are specifically important in regulating the levels of the heart-specific phospholipid cardiolipin [13]. However, only a few investigations have been carried out on the contribution of ether lipids to normal cardiac structure and function or the consequences of ether lipid deficiency for the heart. Reports from medical centers in the Netherlands and North America have concurrently described the frequent occurrence of congenital heart disease in RCDP patients [14,15], but more extensive analyses elucidating the various aspects of pathology and the underlying molecular defects still remain to be performed. Given their important role in membrane biology, ether lipids—in particular, plasmalogens—have manifold potential to impact cardiac development, structure, and activity. For example, membrane proteins like connexin 43 (Cx43), which is essential for electrical coupling and whose levels are strongly reduced in ether-lipid-deficient mouse fibroblasts and cardiac tissue [16,17], may be impacted by an altered phospholipid environment. Furthermore, it has been shown that anomalies in phospholipid-dependent signaling processes are associated with the development of cardiac disease [18]. In addition, data from studies in *Drosophila* underline that the disruption of phospholipid homeostasis has detrimental consequences for cardiac function [19]. Vice versa, diseases of the cardiovascular system often go along with alterations in phospholipid levels. In the case of plasmalogens, reduced levels have, for example, been reported in the plasma of patients affected by hypertension [20].

In the present study, we aim to provide a comprehensive characterization of the cardiac phenotype resulting from ether lipid deficiency at the morphological and functional level and attempt to identify molecular and biochemical alterations in ether-lipid-deficient cardiac tissue that may underlie functional changes. We used an ether-lipid-deficient mouse model, the *glyceronephosphate O-acyltransferase* knockout (*Gnpat* KO) mouse [16], to study cardiac pathology in closer detail and compared the findings to relevant medical data obtained from RCDP patients. While these mice have a complete ether lipid biosynthesis defect, lipid analyses have detected small amounts of plasmalogens presumably originating from the diet in various tissues, including the heart [17]. In addition, lipidomic investigation of the plasma has identified traces of other, non-plasmalogen ether lipids [21], which may also be present in cardiac tissue of *Gnpat* KO mice. Our results reveal a complex cardiac phenotype with species-dependent differences upon ether lipid deficiency and provide further evidence supporting the importance of phospholipid homeostasis for cardiac structure and function.

## 2. Results

### 2.1. Ventricular Septal Defects Are Present in Developing Gnpat KO Mice

**Figure 1:**
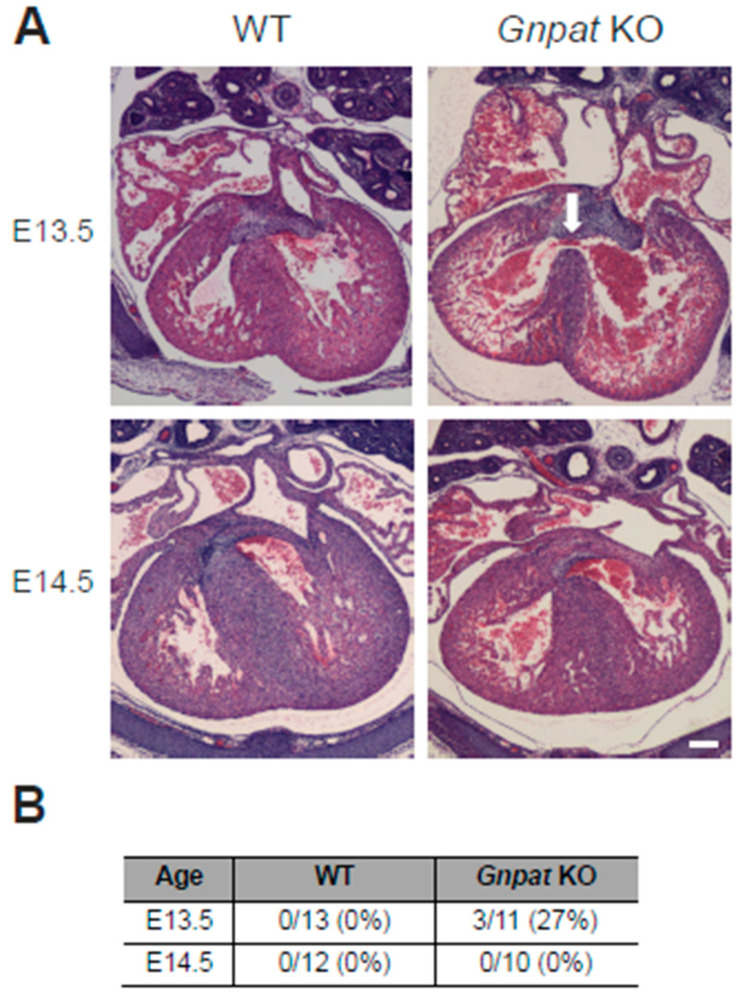
Fetal heart morphology in WT and *Gnpat* KO mice. (**A**) Serial sections (7 µm) of paraffin-embedded WT and *Gnpat* KO fetuses were prepared at two developmental stages (E13.5 and E14.5) and stained with H&E. Representative pictures of transverse sections through the fetal heart with particular focus on ventricular septation are shown. The white arrow (*Gnpat* KO, E13.5) indicates the presence of a ventricular septal defect. Only animals derived from the same litter of heterozygous matings were directly compared. Scale bar = 100 µm. (**B**) Percentages of murine embryos with ventricular septal defect at different embryonic stages.

A major hallmark of the cardiac phenotype in RCDP patients, as described so far [14,15], is the abundance of congenital heart disease, in particular septal defects. To assess whether this phenotype is reproduced in the *Gnpat* KO mouse model, we first evaluated cardiac morphology histologically in developing embryos from heterozygous matings. We retrieved WT and *Gnpat* KO fetuses at E13.5, a developmental stage at which ventricular septation should be completed [22], and at the subsequent stage, E14.5. As judged by H&E-stained serial, transverse sections ranging from the base of the heart to the apex of the ventricles, the ventricular septum fully separated the two ventricles in all WT embryos studied at both time points (E13.5, *n* = 13; E14.5, *n* = 12), as expected (Figure 1). Compared with WT littermates, septation lagged behind in ether-lipid-deficient embryos. The majority of *Gnpat* KO embryos at E13.5 (eight out of eleven, 73%) exhibited a separation of the two ventricles; however, the cell layer making up the ventricular septum appeared thinner than in WT littermates. In several cases (three out of eleven, 27%), we found obvious ventricular septal defects allowing the exchange of blood between the ventricles (Figure 1, upper right panel). The ventricular septal defect was accompanied by transposition of the great vessels, a condition in which the spatial arrangement of the major vessels associated with the heart is altered, resulting in an undersupply of the body with oxygenated blood. Remarkably, evaluating a similar number of animals, we did not identify any ventricular septal defect in *Gnpat* KO fetuses at E14.5 (*n* = 10). In several cases, though, septation appeared incomplete, as separation between the ventricles consisted of only a few layers of myocytes, in contrast to the several hundred µm thick septum in WT animals (Figure 1A, lower panels; summary of the results in Figure 1B). Based on these findings, we hypothesized that the aberrations in cardiac development in ether-lipid-deficient mice reflect a developmental delay. If this assumption were correct, adult ether-lipid-deficient animals would present with a normal septum. We addressed this hypothesis by performing cardiac MRI assessment in a small animal preclinical MRI system. In alignment with our expectations, we did not detect any signs of a ventricular septal defect in *Gnpat* KO mice (*n* = 10) or WT controls (*n* = 11) aged 13.5–15.5 months.

**Figure 2:**
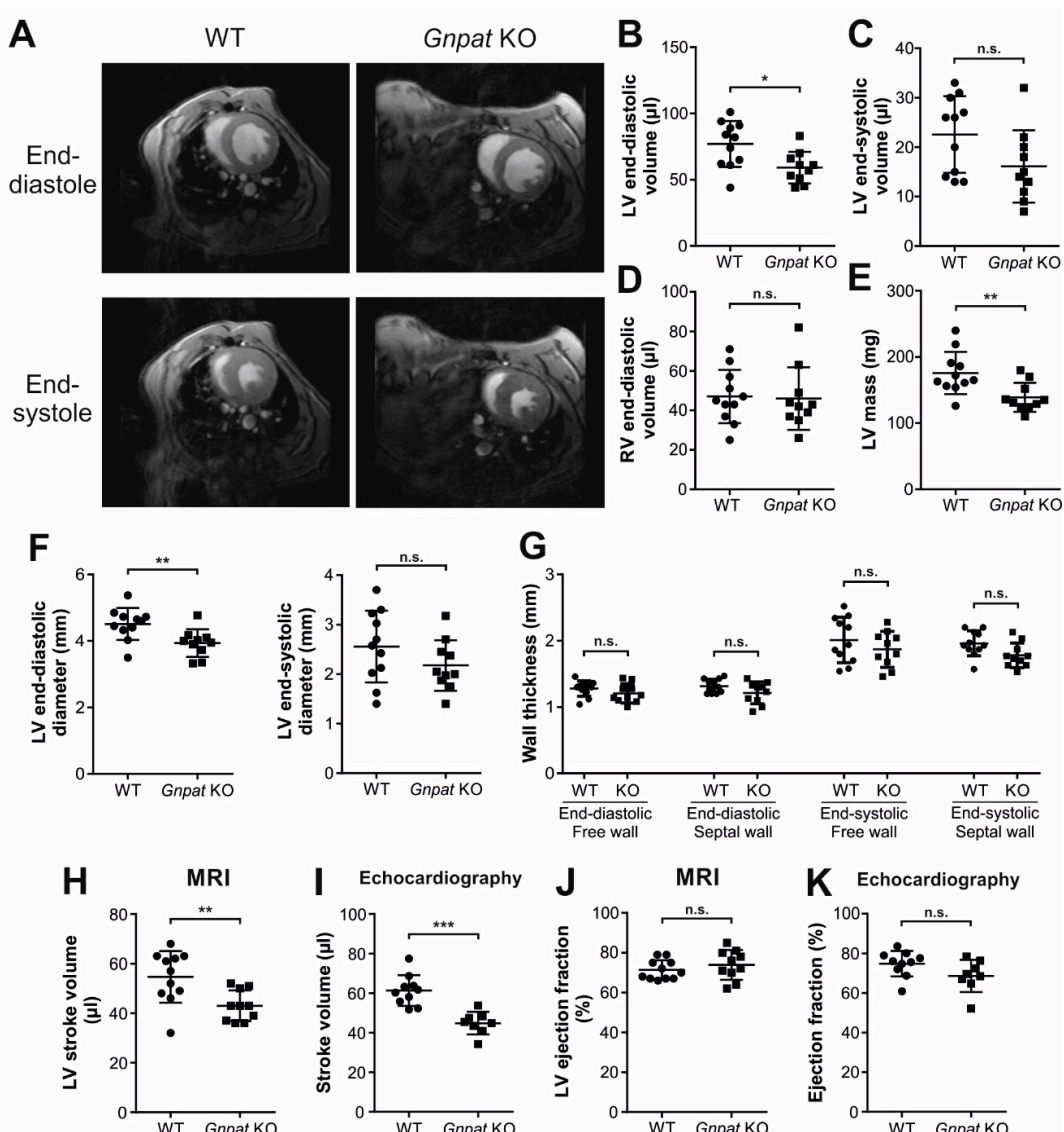
Morphometric and functional analyses of middle-aged WT and *Gnpat* KO mice. (**A**) Representative magnetic resonance imaging (MRI) short axis slice depicting WT and *Gnpat* KO hearts during end-diastole and end-systole. Left ventricle (LV) and right ventricle (RV) are shown. (**B**–**K**) LV end-diastolic volume (**B**), LV end-systolic volume (**C**), RV end-diastolic volume (**D**), LV mass (**E**), LV end-diastolic as well as end-systolic diameter (**F**), wall diameter (**G**), LV stroke volume (**H**), and LV ejection fraction (**J**) were determined by MRI (WT: *n* = 11; *Gnpat* KO: *n* = 10). In addition, stroke volume (**I**) and ejection fraction (**K**) were determined by echocardiography in WT (*n* = 10) and *Gnpat* KO (*n* = 8) mice. Data for individual mice are presented (circles, WT; squares, *Gnpat* KO) together with group means ± SD, and statistical analysis was performed using two-tailed Student’s *t*-tests. Bonferroni–Holm correction for multiple comparisons was applied in the case of wall diameters (panel (**G**)). *** *p* < 0.001; ** *p* < 0.01; * *p* < 0.05; n.s., not significant.

In the same adult cohort, we performed a morphometric analysis of the heart using MRI (for representative images, see Figure 2A). We found decreased end-diastolic volume (EDV) of the LV but not the right ventricle as well as reduced LV mass (LVM) in *Gnpat* KO compared with WT mice (Figure 2B,D,E). However, the difference in LVM disappeared when LVM was related to body weight (LVM/body weight (×10^3^; mean ± SD): WT: 3.73 ± 0.61, *Gnpat* KO: 3.77 ± 0.31; *p* = 0.852 (two-tailed Student’s *t*-test)). End-systolic volume (ESV) was reduced by trend as well, but the difference did not reach statistical significance (*p* = 0.065; Figure 2C). Similarly, ether-lipid-deficient mice showed smaller LV end-diastolic (but not end-systolic) diameter (Figure 2F). Remarkably, we did not detect any statistically significant differences in the thickness of the LV septal or free wall, neither in systolic nor in diastolic measurements (Figure 2G). Moreover, assessment of myocardial thickness in another cohort of similar age by echocardiography revealed no difference in diastolic or systolic wall thickness between WT and KO animals (Supplementary Figure S1A).

### 2.2. In Vivo Assessment of Cardiac Function Reveals Normal Ejection Fraction but Reduced Stroke Volume in Middle-Aged Ether-Lipid-Deficient Mice

Following the morphologic examination of the ether-lipid-deficient heart, we focused on the evaluation of cardiac function in middle-aged *Gnpat* KO mice using MRI. Ether-lipid-deficient mice showed diminished stroke volume compared with WT mice (Figure 2H), whereas the ejection fraction was similar in *Gnpat* KO and WT mice (Figure 2J). To complement these findings by an independent method, we performed echocardiography in mice aged 12–14 months. In line with the results of our MRI experiments, we did not detect any statistically significant differences in ejection fraction and fractional shortening between the genotypes (Figure 2K, Supplementary Figure S1B), but again, stroke volume was clearly reduced in *Gnpat* KO mice (Figure 2I).

### 2.3. Ether-Lipid-Deficient Mice Show Increased Left Ventricular Pressure

**Figure 3:**
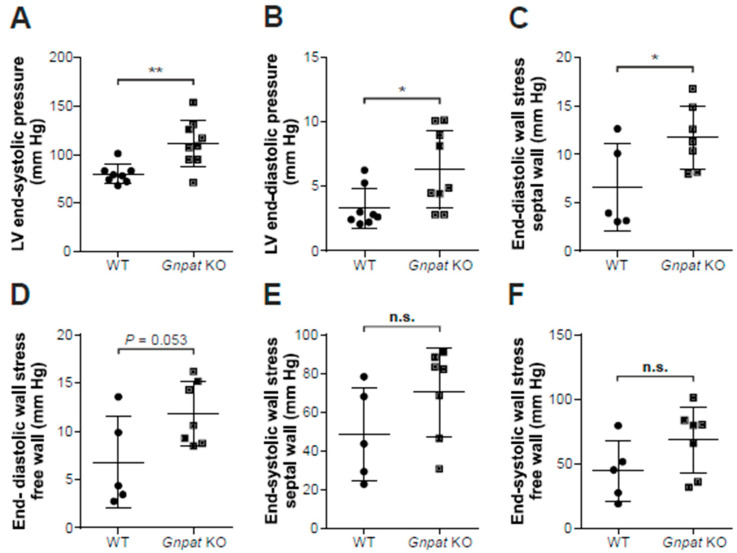
In vivo assessment of hemodynamic parameters in WT and *Gnpat* KO mice. LV end-systolic pressure (**A**) and LV end-diastolic pressure (**B**) were recorded using a microtip catheter inserted into the LV (WT: *n* = 8; *Gnpat* KO: *n* = 9). The MRI wall diameter measurements during end-diastole and end-systole (cf. Figure 2F,G) from the same cohort of mice (WT: *n* = 5; *Gnpat* KO: *n* = 7; note that not every parameter was available for all mice) were used to calculate septal and free ventricle wall stress during diastole (**C** and **D**, respectively) and during systole (**E** and **F**, respectively). Data for individual mice are presented (circles, WT; squares, *Gnpat* KO) together with group means ± SD. Statistical analysis was performed using two-tailed Student’s *t*-tests. ** *p* < 0.01; * *p* < 0.05; n.s., not significant.

In a next step, we investigated LV pressure via insertion of a fine-tipped catheter into the LV. Strikingly, both LV end-systolic and end-diastolic pressure were statistically significantly elevated in 13–16-month-old *Gnpat* KO compared with WT mice (Figure 3A,B). Accordingly, we also noticed an increase in the maximal rate of pressure increase (dP/dt max) in *Gnpat* KO animals (mean ± SD: WT: 4392 ± 957 mm Hg/sec, *n* = 8; KO: 5541 ± 1204 mm Hg/sec, *n* = 9; *p =* 0.048, two-tailed Student’s *t*-test). No alterations, however, were found in the maximal rate of pressure decay (dP/dt min; mean ± SD: WT: −3508 ± 1304 mm Hg/sec, *n* = 8; KO: −4247 ± 1178 mm Hg/sec, *n* = 9; *p =* 0.238, two-tailed Student’s *t*-test). Using the dimension data gathered in MRI investigations for the same group of mice, we calculated the wall stress acting on the myocardium of WT and *Gnpat* KO animals. Notably, when comparing ether-lipid-deficient mice against WT, wall stress at end-diastole was significantly increased at the septal wall (Figure 3C) and almost reached statistical significance for the free ventricle wall (*p =* 0.053; Figure 3D). However, there was no statistical difference in wall stress between the genotypes at end-systole (Figure 3E,F). Furthermore, heart rates were similar in WT and *Gnpat* KO mice (mean ± SD: WT: 336 ± 62 bpm; Gnpat KO: 332 ± 19 bpm; *p* = 0.878 (two-tailed Student’s *t*-test)). The results of all measured cardiac parameters in adult mice are summarized in Table 1.

### 2.4. No Overt Cardiac Fibrosis in Gnpat KO Mice

Since wall stress is a frequent cause for ventricular hypertrophy, we systematically recorded the ratio between heart weight and body weight (HW/BW) in all WT and *Gnpat* KO mice used in our laboratory. Interestingly, there was a modest, but statistically highly significant increase in the HW/BW ratio of *Gnpat* KO compared with WT mice (HW/BW (×10^3^) ± SD: WT: 4.577 ± 0.789, *n* = 102; KO: 5.187 ± 0.925, *n* = 86; *p* < 0.001 (two-tailed Student’s *t*-test)), which may be an indicator of hypertrophy. Notably, hypertrophy is comparatively frequently observed in RCDP patients, for example, in the context of tetralogy of Fallot (cf. Section 2.6). However, ether-lipid-deficient mice, particularly at higher age, are almost completely devoid of white adipose tissue resulting in reduced body weight ([23] and own unpublished observation), which might be an influential factor in the alteration of the HW/BW ratio as well. In order to investigate whether myocardial fibrosis resulting in hypertrophy of the LV could play a role in the cardiac phenotype of ether-lipid-deficient mice, we applied several histological staining techniques (H&E, Masson–Goldner trichrome, Sirius Red, and van Gieson) on cardiac tissue from middle-aged (13 months) WT and *Gnpat* KO mice. However, the extent of fibrosis was low in both genotypes, and no difference was observed between WT and *Gnpat* KO tissue using these staining assays (*n* = 5/genotype; for representative pictures, see Supplementary Figure S2A–D). Confirming the histological findings, quantitative real-time PCR analysis of heart homogenates did not identify any differences in mRNA levels between WT and *Gnpat* KO samples (age 12.5–16 months) in the connective tissue markers collagen I (*Col I*) and collagen III (*Col III*) or the hypertrophy indicators atrial natriuretic peptide (*Anp*), B-type/brain natriuretic peptide (*BNP*), and myosin heavy chain 7 (*Myh7*) (Supplementary Figure S2E).

### 2.5. Ether Lipid Deficiency Leads to Compromised Cardiac Impulse Conduction

In a recent study, we demonstrated reduced protein levels of Cx43 in cardiac tissue homogenates of aged (14–16.5 months) *Gnpat* KO mice [17]. However, in the present work, contrary to our expectations, immunohistochemical detection in situ revealed a normal assembly of Cx43 in intercalated discs, structures formed at the junctions between cardiomyocytes, of the myocardium (Supplementary Figure S2F,G).

Furthermore, based on our earlier findings of synaptic loss of several neurotransmitters, particularly monoamines, in the brain of ether-lipid-deficient mice [24] and the fact that the heart receives noradrenergic input from peripheral neurons originating from sympathetic ganglia, we also analyzed neurotransmitter levels in the heart. Therefore, we prepared blood-free homogenates of cardiac tissue derived from aged WT and *Gnpat* KO mice and determined the levels of norepinephrine and dopamine. As in brain tissue, the amounts of norepinephrine were markedly reduced, by more than 23%, in cardiac tissue from *Gnpat* KO mice as compared with WT mice (Supplementary Figure S3A). Interestingly, this depletion did not only affect the levels of norepinephrine but—even more pronouncedly (−50%)—also those of dopamine (Supplementary Figure S3B), which, however, is only present in much lower amounts in the mammalian heart [25,26].

**Figure 4:**
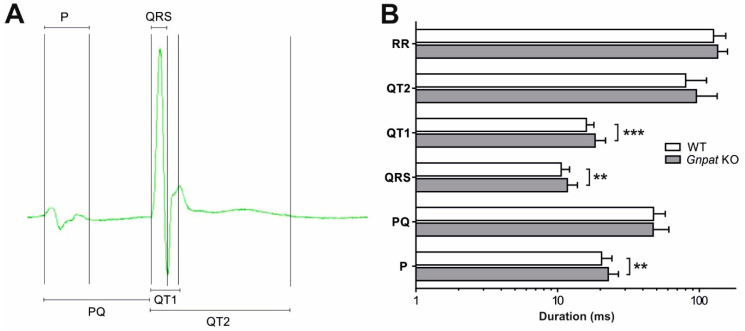
Resting ECG analysis of WT and *Gnpat* KO mice. (**A**) Schematic overview of the analyzed ECG intervals. (**B**) WT (*n* = 45) and *Gnpat* KO (*n* = 40) mice were exposed to resting ECG examinations, and the results for each interval are presented as mean ± SD. RR indicates the interval between two neighboring R peaks, i.e., the average duration of one heartbeat. Statistical analysis was performed using two-tailed Student’s *t*-tests. *** *p* < 0.001; ** *p* < 0.01.

The reduction in Cx43 levels [17] and the depletion of norepinephrine as well as the profound morphological and mechanical differences between WT and *Gnpat* KO mice all have the potential to impact the cardiac conduction system. We previously determined key parameters of ventricular conduction in a small cohort of mice and showed that a conduction deficit can be rescued by treatment with ether lipid precursors [17]. Here, we extend our ECG investigations considerably with the aim of providing a comprehensive characterization of cardiac transmission upon ether lipid deficiency. In resting ECG recordings (for an overview of analyzed intervals, see Figure 4A), the duration of both the P wave and the QRS complex was significantly increased in KO animals (Figure 4B), indicating a decrease in conduction velocity in the atria and ventricles due to ether lipid deficiency. QT1 values were also increased in KO animals, which may be secondary to the increase in QRS duration. Our experimental cohort contained animals of different age groups, and a more detailed analysis of the data demonstrated a significant positive correlation between age and QRS duration in *Gnpat* KO but not in WT mice (Supplementary Figure S4), suggesting that impaired conduction in ether-lipid-deficient animals is age-dependent. Furthermore, the ratio between the amplitudes of the R and S waves was substantially lowered in the KO animals (Table 2). KO animals also showed a significantly increased dispersion of both QRS and QT1 intervals (Table 2), possibly indicating a decrease in the spatial homogeneity of ventricular activation and repolarization in *Gnpat* KO compared with WT mice.

**Figure 5:**
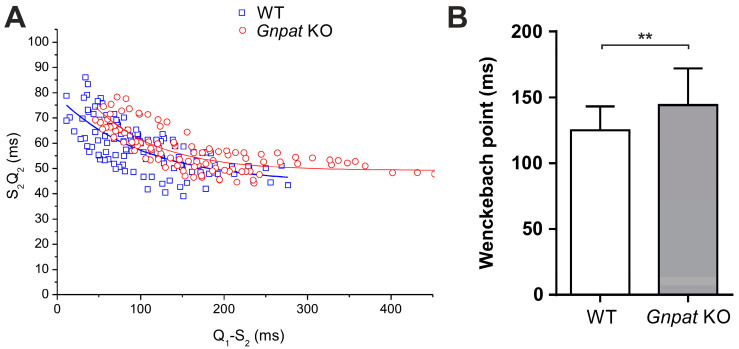
Frequency-dependent differences between WT and *Gnpat* KO mice upon transesophageal stimulation. Continuous transesophageal stimulation in WT and *Gnpat* KO mice was used to study the effect of atrial pacing on AV-nodal conduction. (**A**) AV conduction time (S2Q2) is shown as a function of the interval between the preceding Q wave and the following stimulus artefact (Q1S2). At least three data points were acquired for each individual mouse (WT: *n* = 11; *Gnpat* KO: *n* = 9). Curves were fitted using a monoexponential function as described in Material and Methods. (**B**) Wenckebach cycle length was determined in WT (*n* = 27) and *Gnpat* KO (*n* = 23) mice and is depicted as mean ± SD. Statistical analysis was performed using two-tailed Student’s *t*-test. ** *p* < 0.01.

In addition to indicators of ventricular conduction and repolarization, we sought to examine whether conduction properties of the AV node are altered in *Gnpat* KO animals. Because AV-nodal conduction depends on the heart rate, it was necessary to determine AV-nodal conduction times at defined heart rates generated by transesophageal programmed stimulation. Here, although the curves fitted to the rate-dependent values for S2Q2 were not statistically different between genotypes, the curve determined in *Gnpat* KO animals appeared to be shifted to greater S2Q2 values, especially at lower pacing cycle lengths (Figure 5A). Furthermore, S2Q2 values could be determined at shorter Q1S2 intervals in WT animals than in KO animals. Accordingly, the Wenckebach point (the minimum cycle length needed to maintain 1:1 AV conduction) was significantly increased in *Gnpat* KO mice, indicating depressed AV-nodal function (Figure 5B). An overview of all functional tests targeting cardiac parameters in ether-lipid-deficient mice is provided in Table 1.

### 2.6. Human RCDP Patient Data Indicate a Complex Cardiac Pathology upon Ether Lipid Deficiency

Cardiac pathology in human ether-lipid-deficient patients is complex and common. Previous studies demonstrated the frequent occurrence of congenital heart disease in patients with severe but also the less severe forms of RCDP (Table 3). Adding to these data, we retrieved echocardiography data from a centralized registry of RCDP patients, most of whom have been reported in earlier publications [14,15,27], and provide the detailed echocardiography findings, also including four previously uncharacterized patients in Supplementary Table S1. The summarized findings confirm a wide range of pathologies, including ventricular and atrial septal defects, conotruncal anomalies, and congenital mitral valve prolapse. While none of the pathological features is fully penetrant in children with RCDP, septal defects are the most common denominator in patients with cardiac pathology (cf. Table 3). In addition, we analyzed hemodynamic data available from the RCDP registry. Blood pressure was largely normal in a total of 57 measurements recorded from 14 patients (Supplementary Table S1). Heart rates were found appropriate to age in most examinations but were elevated for some of the patients at individual time points (Supplementary Table S1).

To investigate if defects in cardiac transmission, as observed in the mouse model, are also reflected in RCDP patients, we performed ECG recordings in 10 RCDP type 1 patients attending an annual meeting. Results are presented in Table 4. Unlike in the murine cohort, ECG conduction delays were not identified in this patient series. On the contrary, three patients were found to have an unusually short corrected QT (QTc) interval [28]. The remaining seven patients had QTc intervals within the normal range. Of note, in all patients, the QTc was ≤ 400 ms. Most patients had an rSR’ complex, i.e., another R wave following the S wave of the QRS complex, in the V1 lead, which may be indicative of an underlying atrial septal defect. The remainder of the ECG recordings were unremarkable regarding PQ and QRS intervals.

## 3. Discussion

The present characterization of the cardiac phenotype under conditions of ether lipid deficiency adds substantially to our understanding of ether lipids in physiology and pathophysiology. To our knowledge, this analysis represents the first systematic report of cardiac pathology in ether-lipid-deficient mice. Specifically, we demonstrate delayed ventricular septum formation, reduced stroke volume with preserved ejection fraction, increased wall stress, and slower ventricular conduction, as well as impaired AV-nodal function in *Gnpat* KO mice. Furthermore, we have summarized and extended previously reported characteristics and manifestations of cardiac pathology in human patients with inherited ether lipid deficiency (RCDP) in order to identify phenotypic similarities and differences between human disease and the animal model, with the final aim to better understand the complex interrelation between a specific lipid defect and cardiac morphology and function.

Deficiency in ether lipids, particularly plasmalogens, has complex consequences for the lipid environment and, thus, the biophysical properties of biological membranes. As we have shown in a recent study, the almost complete deficiency of ethanolamine plasmalogens in cardiac tissue of *Gnpat* KO mice is accompanied by a strong rise in the levels of the diacyl lipid phosphatidylethanolamine (PE) [17]. All other main phospholipid classes—including phosphatidylcholine, for which no compensatory effect was observed—remained unchanged upon ether lipid deficiency. These findings were in line with our previous work suggesting a regulatory mechanism, by which PE levels are strictly adapted to the levels of ethanolamine plasmalogens [30]. While the phenotype upon ether lipid deficiency is typically attributed to the lack of ether lipids, the inevitable increase in PE levels may also lead to cellular disturbances, for example, by influencing membrane integrity and rigidity [31,32]. As we have deliberated previously, when considering the asymmetric distribution of the different phospholipid classes in membranes, the unequal compensatory mechanisms between choline and ethanolamine phospholipids could be detrimental [7]. Accordingly, the molecular alterations resulting in the cardiac phenotype of *Gnpat* KO mice may be of a dual nature caused by the deficiency of ether lipids on the one hand and the accumulation of PE on the other hand.

Since septal defects are regularly observed in RCDP patients [14,15], we here decided to investigate the occurrence of ventricular septal defects in developing and adult *Gnpat* KO mice and whether these are direct consequences of ether lipid deficiency. Remarkably, we found an abnormally open septum in 27% of ether-lipid-deficient fetuses at developmental stage E13.5 but not at later time points (E14.5 or adult), suggesting a delay of cardiac development in these animals. Retarded development in ether-lipid-deficient animals has also been reported previously in the context of cerebellar structures [33]. Indeed, “plasmalogen metabolism” has been identified as one of the key metabolic pathways enriched in neonatal mouse hearts, thus supporting an essential role in cardiac development [34]. Although the molecular mechanisms governing ventricular septation are still obscure [35], plasmalogens could impact the process by their proposed role as antioxidants and as essential components of lipid membranes. Furthermore, signaling cascades play critical roles in the closure of the septum and are, as we have summarized recently, markedly influenced by a lack of plasmalogens and other ether lipids [8]. Actually, subtle alterations in different signaling processes might amplify each other, resulting in widespread disturbances and inducing developmental aberrations. However, we cannot fully rule out the idea that fetuses with a ventricular septal defect at E13.5 are simply abandoned during further development and, thus, cannot be detected at later developmental stages. Strikingly, *Gnpat* KO mice are not born in strictly Mendelian ratios but make up only 15–20% of the offspring of heterozygous parents at early postnatal stages ([16] and personal observation). This raises speculation as to whether the ventricular septal defect in a subset of embryos leads to prenatal or early postnatal death, thereby accounting for the “lost” proportion of *Gnpat* KO newborns. However, at least in humans, a ventricular septal defect is not necessarily a life-threatening condition [36], and it has to be considered that *Gnpat* KO mice suffer from other conditions impacting pre- and postnatal survival [16,37]. Interestingly, mice deficient in the protein tafazzin, an important contributor to lipid remodeling, presented markedly reduced levels of choline plasmalogens in the heart [13] along with abnormal septation [38]. Furthermore, the tafazzin-deficient animals present with cardiomyopathy due to a defect in early development [38]. However, the underlying mechanisms in this model are presumably complex and may involve altered distribution of the acyl species in cardiolipins in addition to the plasmalogen deficit. Moreover, a mouse strain with cardiac-specific overexpression of Mammalian Sterile 20-Like Kinase 1 (Mst1/Stk4) develops cardiomyopathy and has reduced plasmalogen levels, which was hypothesized to play a causative role for the cardiac defect [39]. However, the involvement of plasmalogens in the pathologic process remains unclear, since in that mouse model a variety of metabolic and cellular alterations are likely, and supplementation with an ether lipid precursor could not improve cardiac function [39].

Nevertheless, these observations prompted us to investigate cardiac mechanical function in adult *Gnpat* KO mice. The ejection fraction and fractional shortening, as evaluated by two independent measurements via MRI and echocardiography, were normal, suggesting that ether lipid deficiency alone is not sufficient to provoke cardiomyopathy. Yet, whereas wall diameters did not differ between WT and ether-lipid-deficient animals, our morphometric MRI analyses showed reduced LV end-diastolic volume, end-diastolic diameter, end-systolic diameter, and mass, along with a lower stroke volume in *Gnpat* KO mice. However, the changes in these parameters may well be a result of the growth retardation of *Gnpat* KO mice, which leads to the smaller size of several organs, as we previously showed in the case of the brain [24]. The depletion of norepinephrine in ether-lipid-deficient mice may further contribute to these observations considering that, when applied exogenously in humans, norepinephrine increases stroke volume and cardiac output [40,41].

In a previous study focusing on therapeutic strategies against cardiac pathology in ether lipid deficiency [17], we already demonstrated prolonged QRS duration upon ECG examination of *Gnpat* KO mice. Here, we confirmed these observations in an expanded cohort, thus further emphasizing the defect in ventricular conduction that particularly affects aged ether-lipid-deficient mice. Based on Western blot data, we speculated that this defect stems from reduced levels of Cx43 [17], the major component of ventricular gap junctions. Accordingly, Cx43 is a critical determinant of cardiac impulse conduction, and its altered expression and/or malfunction has been shown to trigger severe cardiac conduction defects in mice and dogs [42,43,44]. Under conditions of ether lipid deficiency, the altered membrane lipid environment may lead to decreased stability of membrane proteins like Cx43 or interfere with the function of ion channels [45], which are main players in the cardiac transmission of electrical signals. Next to the parameters characterizing ventricular conduction (QRS, QT1), also the P wave, an indicator of atrial depolarization, was significantly longer in *Gnpat* KO mice. Notably, Cx43 is prominently expressed in the atrium [46], and Cx43 deficiency has been reported to result in delayed electrical impulse propagation in atrial cardiomyocytes [47]. Interestingly, reduced Cx43 levels have also been associated with tetralogy of Fallot [48], a condition repeatedly observed in RCDP patients. However, there, like in other cardiac diseases characterized by abnormal Cx43 [49], the spatial distribution of Cx43 was also affected [48], whereas in the ether-lipid-deficient murine heart, the assembly of Cx43 in intercalated discs appeared unaltered. Finally, impulse conduction across the AV node may also be compromised in *Gnpat* KO mice, as indicated by the increased Wenckebach point and the “upward” displacement of rate-dependent AV-nodal conduction time, when compared with WT mice. To characterize intraventricular conduction in detail, we also focused on less commonly investigated parameters from ECG readings. Notably, ether-lipid-deficient mice exhibited a markedly decreased R/S amplitude ratio. Similar changes have been observed in patients suffering from Duchenne muscular dystrophy and in murine models of the disease [50,51] and were proposed to reflect alterations in the pathways of ventricular conduction as a result of fibrosis, altered ventricular wall thickness, or regional functional inhomogeneity in cellular electrophysiological properties. However, our results from histology, MRI, and echocardiography analyses negate the presence of fibrosis or altered ventricular wall thickness in *Gnpat* KO mice, thus leaving regional differences in electrophysiological characteristics stemming from the abnormal lipid environment as the most likely explanation for the reduced R/S amplitude. Furthermore, we identified increased dispersion of both QRS and QT1 intervals in ether-lipid-deficient mice. Dispersion, which is defined as the difference between the maximal and the minimal values of interval duration in the various leads, quantifies the extent of inhomogeneity in ventricular activation and/or repolarization [52]. Abnormal dispersion has been associated with increased arrhythmic events and mortality in humans [53,54,55,56] and has also been found in murine models of arrhythmogenic heart disease [57,58], as well as in adult mice with an induced deletion of Cx43 [42]. Furthermore, an impairment of ion channels resulting from the perturbed lipid environment in ether lipid deficiency has hard-to-predict consequences for electrical signal transmission and may well cause regional imbalances in cardiac conduction [45].

In addition to alterations in Cx43 (as mentioned above), the depletion of norepinephrine in cardiac tissue could be the basis for the observed trend toward prolonged AV-nodal conduction times in *Gnpat* KO mice. Indeed, pharmacologic autonomic blockade produces prolongation of rate-dependent AV-nodal conduction times [59]. Similar to norepinephrine, the levels of dopamine were strongly reduced in ether-lipid-deficient cardiac tissue. However, one must consider that because the heart contains only few dopaminergic synapses, dopamine predominantly serves as a precursor of norepinephrine. Thus, it is tempting to speculate that the dopamine reduction in the heart of *Gnpat* KO animals is caused by an increased activity of dopamine ß-hydroxylase in response to reduced norepinephrine levels. At the same time though, a vesicular uptake defect, like we hypothesized previously for the brain [24], represents a likely explanation for the reduction in both dopamine and norepinephrine as well.

Interestingly, the findings of delayed ventricular conduction in the mouse model could not be reproduced in our series of RCDP patients. Instead, three out of ten RCDP patients studied by ECG showed QTc intervals of ≤ 0.34 s, the diagnostic criterion for short QT syndrome [60], even though in pediatric cohorts, other factors should also be considered [61]. Given the low prevalence of a short QT interval in the general population, with QTc below 340 ms observed in only 0.4% [62], these findings warrant further investigation for the clinical evaluation of RCDP. Short QT syndrome can cause arrhythmia with sudden cardiac death but can also be asymptomatic. In previous studies, these disorders have been characterized as channelopathies caused by mutations in genes encoding Na, Ca, or K channels [29]. Interestingly, defects in some of the described genes also cause long QT syndromes. Of note, a direct influence of ether lipids on ion channels has been hypothesized previously [45], but the consequences of ether lipid deficiency for ion channel function have yet to be investigated. However, ion channel expression, properties, and function can differ considerably between humans and animal models [63]. Thus, it is conceivable that ion channels are the determining factor in the changes that we observed in ECG recordings in both mice and humans.

## 4. Material and Methods

### 4.1. Human Patient Data

De-identified data from patients diagnosed with RCDP were retrieved from the Rhizomelic Chondrodysplasia Punctata registry (maintained at the Skeletal Dysplasia Center, Nemours Children’s Hospital, Wilmington, DE, USA (ClinicalTrials.gov Identifier: NCT04569162)). In total, we obtained clinical information from 18 different patients. Hemodynamic data (blood pressure and heart rate) were available for 15 patients, and 33 echocardiography reports from 15 different patients were screened. Data from some of these patients were described before [15,27]. Portable 12-lead electrocardiograms for 10 RCDP patients were collected at the annual RhizoKids meeting, Alexander City, AL, USA in July 2019. Informed consent was obtained through the Longitudinal Natural History Study of Patients with Peroxisomal Disorders (Research Institute of the McGill University Health Center, Montreal, QC, Canada (ClinicalTrials.gov Identifier: NCT01668186)).

### 4.2. Mice

Mice with a targeted inactivation (knockout, KO) of the *Gnpat* gene (*Gnpat^tm1Just^*) have been described previously [16]. The strain was maintained on an outbred C57BL/6 × CD1 background, and experimental cohorts with *Gnpat*^−/−^ (KO) and *Gnpat*^+/+^ (wild type, WT) littermates were obtained by mating heterozygous animals. Genotypes were determined at weaning by polymerase chain reaction (PCR) as described previously [16] and confirmed after sacrifice. Mice were fed standard chow with food and water ad libitum and were housed in a temperature- and humidity-controlled room with a 12:12 h light–dark cycle and a low level of acoustic background noise at the local animal facility of the Medical University of Vienna. In all experiments, age- and sex-matched WT animals, from the same litters when possible, were used as controls to minimize variability, except for the experiments involving embryonic tissue, in which sex was not determined.

Experiments were carried out in compliance with the 3Rs of animal welfare (replacement, reduction, refinement), and the number of animals was reduced to the estimated minimum necessary to obtain clear-cut, statistically significant results. Required approval for individual experiments was obtained from the Institutional Animal Care and Use Committee of the Medical University of Vienna and the Austrian Federal Ministry of Education, Science and Research (BMWFW-66.009/0147-WF/II/3b/2014 and BMBWF-66.009/0174-V/3b/2019 to J.B., BMWF-66.009/0211-II/10b/2009 to R.E.B. and GZ 0278-II/3b/2012 to B.K.P.).

### 4.3. Magnetic Resonance Imaging and Post-Processing

Magnetic resonance imaging (MRI) was performed on a 9.4 Tesla Biospec 94/30 USR system (Bruker Biospin, Ettlingen, Germany) with maximal achievable gradient strength of 667 mT/m. A gradient insert with inner diameter of 116 mm was used. For radiofrequency excitation, a transmitter volume resonator with an inner diameter of 86 mm was utilized; image acquisition was conducted using a dedicated mouse heart coil array with four elements. Mice were pre-anesthetized with isoflurane and positioned on a dedicated heated mouse bed. Inhalative anesthesia was maintained with a mix of 1.5–2% isoflurane and oxygen by face mask. Respiration, heart rate, and body temperature were monitored throughout the examination. Isoflurane levels were adjusted according to the respiration rate.

A prospective electrocardiography (ECG)-gated cine gradient echo-based flow-compensated MR sequence implemented in the in-built software ParaVision 6.0 (Bruker Biospin, Ettlingen, Germany) was acquired for cardiac function assessment. An average of ten consecutive slices were acquired along the long axis covering the entire heart volume from the apex to the base. The following imaging acquisition parameters were used: time of echo (TE) = 2.4 ms, time of repetition (TR) = 8 ms, number of signal averages (NSA) = 6, field-of-view (FOV) = 25 mm × 25 mm, slice thickness (ST) = 0.8 mm, flip angle (FA) = 15°, partial Fourier transformation (pFT) = 1.45, measured matrix size = 132 × 192, reconstructed matrix size = 192 × 192, and movie frames (MF) = 18.

Left ventricle (LV) analysis was carried out using freely available software (Segment Software, v1.8 R1172; Medviso AB, Lund, Sweden) [64], as described previously [65]. The cine sequence was used to determine the end-systole and end-diastole of the heart action. The end-systolic and end-diastolic volumes (ESV and EDV, respectively, in ml) were measured by manual segmentation of the LV on each axial slice excluding the papillary muscles. The ejection fraction (EF% = (EDV − ESV)/EDV) was calculated automatically. For each mouse, LV diameter at end-systole and end-diastole, as well as wall thickness determinations, were derived from the average of three to five independent measurements.

### 4.4. Echocardiography

Mice were anesthetized using chloral hydrate (35 mg/mL in 150 mM NaCl) applied intraperitoneally (0.1 mL/10 g body weight). Their ventral thorax was shaved, and mice were fixed on a heating plate (38 °C) to stabilize body temperature. For monitoring reasons, electrodes were inserted subcutaneously to record electrocardiograms. Commercially available echocardiography gel was applied to the thorax of the test animal, and echocardiography measurements were carried out using a Vevo 770 High Resolution In vivo Micro-Imaging System (VisualSonics, Toronto, ON, Canada; ultrasound head: RMV 707B). The optimal plane for measurements was identified in B mode, and final pictures were taken in M mode. At minimum, two pictures were taken for both long and short axis, and the results were averaged for each mouse. For analysis, ventricular walls were tracked manually using the wall trace measurement tool of the VisualSonics software. Using the automated computing function of the program, standard cardiac performance parameters (ejection fraction, fractional shortening, and stroke volume) were calculated automatically.

### 4.5. Assessment of Left Ventricular Hemodynamic Function In Vivo

Following cardiac MRI, hemodynamic parameters were assessed in vivo. Mice were anesthetized by intraperitoneal injection of a mixture of ketamine (100 mg/kg) and xylazine (4 mg/kg), and analgesic therapy was applied subcutaneously (buprenorphine: 0.12 mg/kg body weight). The thorax was opened and a microtip catheter (SPR-1000, Millar Instruments, Houston, TX, USA) inserted into the left ventricular chamber. Hemodynamic parameters such as left ventricular systolic pressure, left ventricular end-diastolic pressure, heart rate, and left ventricular contractility performance (dP/dt) were continuously registered on Labchart (v7.3.2, Powerlab System (8/30), both AD Instruments, Spechbach, Germany). In addition, both systolic and diastolic wall stress were computed accordingly: LV pressure × LV radius/(2 × wall thickness) [66]. The LV radius and LV wall thickness were assessed by cardiac MRI.

### 4.6. Electrocardiography

**Surface electrocardiographic study.** Mice were anesthetized as described above. Using needle electrodes, standard limb leads I-III, aVR, aVL, and aVF were recorded by means of a model 11 G412301 amplifier (Gould Inc., Cleveland, OH, USA). The ECG signals were digitized at 10 kHz (NI LabVIEW SignalExpress, National Instruments, Austin, TX, USA) and averaged over 50 beats. ECG time intervals were determined by calculating the mean of all six standard leads. The PR interval was measured from the beginning of the P wave to the beginning of the QRS complex. To avoid contamination of signals representing impulse conduction with signals representing the early phase of murine cardiac repolarization, the duration of the QRS interval was measured from the sharp onset to the peak of the S wave. The repolarization of the murine cardiac action potential occurs in a rapid and a slow phase [67]. It has been shown that a wave immediately following the S wave contains substantial amounts of early repolarization (“b” wave [68,69,70]). Therefore, to assess the onset of early repolarization, the interval from the Q wave to the peak of the “b” wave was measured (QT1). The duration of final repolarization was assessed by means of the usual QT interval (QT2). The dispersion of ECG time intervals was defined as the maximum difference of the respective values obtained in the six ECG leads.

**Transesophageal electrophysiological study.** For the transesophageal electrophysiological study, a 2F pacing catheter with two 1 mm ring electrodes and an interelectrode distance of 2 mm were used. Bipolar atrial pacing was performed with twice the diastolic threshold (Model 5328 programmable stimulator, Medtronic, Dublin, Ireland). Initial pacing was performed for 60 s at the minimum cycle length that was required to maintain 1:1 atrioventricular (AV) conduction (Wenckebach point). Thereafter, the pacing protocol was repeated with consecutive increases of the pacing cycle lengths by 5 ms until the spontaneous sinus cycle length was reached. AV conduction time during stimulation was defined as the interval between the stimulus artefact (S2) and the following Q wave (S2Q2 interval). For the assessment of the rate dependence of AV conduction, the S2Q2 intervals obtained at different pacing cycle lengths were plotted as a function of the interval between the preceding Q wave and the following stimulus artefact (Q1S2 interval). The data points in this plot were fitted with a mono-exponential function S2Q2 = A*exp(−Q1S2/τ) + S2Q2inf, where S2Q2inf indicates the Q1S2 at infinitely large Q1S2 intervals, A indicates the maximal increase in S2Q2 at Q1S2 = 0, and τ determines the dynamics of the adaptation of S2Q2 intervals to changes in Q1S2.

For each pacing cycle length, sinus node recovery time was determined by measuring the interval between the last stimulus spike and the first spontaneous atrial depolarization after termination of pacing. In addition, the first spontaneous cycle length after sinus node recovery time was determined. In a subgroup of mice, atrial vulnerability was tested by high-frequency atrial burst stimulation for 10 to 12 s, with a pacing cycle length of 25 ms. Burst pacing was repeated 10 times in each animal. Occurrence and duration of inducible arrhythmias were documented.

**Electrocardiography on RCDP patients.** Twelve-lead electrocardiograms (frontal and precordial leads) were conducted by a certified nurse using a portable ECG machine with correct body placement [71]. Electrocardiograms were read by a single cardiologist. QT intervals corrected for heart rate (QTc) were manually calculated using the tangent method and the Bazett formula [72]. Three consecutive QTc intervals were calculated per patient and then averaged. In cases of marked sinus arrhythmia, the QTc was calculated by including the QTc measurements with the longest and shortest RR interval.

### 4.7. Histology and Immunohistochemistry

For myocardial histology of adult mice, hearts were explanted, washed, and perfused with phosphate-buffered saline (PBS), then fixed in a 4% paraformaldehyde (PFA) solution for 24 h. After embedding in paraffin wax, tissue sections (5 µm) were stained with hematoxylin and eosin (H&E), Masson–Goldner trichrome, and van Gieson’s and Sirius Red stains.

For H&E staining of fetuses, dams from staged pregnancies were sacrificed by CO_2_ inhalation and fetuses were isolated, washed in Soerensen’s phosphate buffer (PB; 40 mM NaH_2_PO_4_; 160 mM Na_2_HPO_4_; pH 7.4), and fixed in 4% PFA (in PB). Caudal tissue was removed for genotyping of each fetus before the immersion fixation. After 1 h, the head and lower body were removed, and the residual trunk was incubated in 4% PFA overnight at 4 °C and subsequently embedded in paraffin. Serial sections (7 µm) were prepared, and every fifth was mounted and stained with H&E.

Immunohistochemical staining was performed as described previously [73] with slight adaptations. Mice were perfused transcardially with PB, followed by 4% PFA (in PB), and the hearts isolated, post-fixed in 4% PFA, washed in PB, and embedded in paraffin wax. Tissue sections (5 µm) were deparaffinized in xylene and incubated in methanol containing 0.2% hydrogen peroxide (H_2_O_2_) for 30 min to block endogenous peroxidase activity followed by rehydration in a descending ethanol series. For antigen retrieval, the slides were steamed for 1 h in EDTA buffer (500 µM Tris-Cl, 50 µM EDTA, pH 8.5). After blocking with 10% fetal calf serum (FCS; Lonza, Basel, Switzerland) in Dako buffer (Agilent Technologies, Santa Clara, CA, USA), the primary antibody (rabbit anti-connexin 43, Abcam, Cambridge, Great Britain; cat.no. ab52488; 1:5000 in 10% FCS) was applied overnight at 4 °C. On the next day, tissue sections were incubated in secondary antibody solution (biotinylated donkey α-rabbit, Jackson ImmunoResearch, West Grove, PA, USA; cat.no. 711-065-152; 1:2000 in 10% FCS) and avidin-horse radish peroxidase (Sigma-Aldrich, St. Louis, MO, USA; cat.no. A3151; 1:100 in 10% FCS) both for 1 h at room temperature. Cx43 staining was developed using 3,3′-diaminobenzidine tetrahydrochloride hydrate (DAB; Sigma-Aldrich, St. Louis, MO, USA). Development was stopped by washing with tap water after control of staining progress at the microscope.

### 4.8. Real-Time Quantitative PCR

Total RNA was isolated from LV tissue samples from WT and *Gnpat* KO mice using the RNeasy Mini kit (Qiagen, Venlo, The Netherlands) according to the manufacturer’s instructions. cDNA was prepared using the QuantiTect reverse transcription kit (Qiagen, Venlo, The Netherlands). Samples were analyzed in duplicate in a volume of 20 μL per well. The initial denaturation step of 15 min at 95 °C was followed by 45 cycles of 15 s at 95 °C, 30 s at 50 °C, and 30 s at 72 °C using Rotor-Gene Q software (Qiagen, Venlo, The Netherlands) for Ct value analysis. Relative gene expression was calculated by the 2^−ΔΔCt^ method.

### 4.9. Determination of Monoamines

Mice were sacrificed by cervical dislocation and hearts isolated, weighed, frozen in liquid nitrogen, and stored until further processing at −80 °C. Residual blood was removed from hearts by cutting and washing in ice-cold PBS prior to freezing. Thawed pieces of tissue were homogenized using a tissue homogenizer (Polytron PT3100 equipped with a PT-DA 3012/2 S dispersing aggregate, Kinematica, Malters, Switzerland; 10 s at 15,000 rpm) in 0.1 M perchloric acid (20 volumes) containing 0.4 mM sodium bisulfite (NaHSO_3_). Monoamine neurotransmitters and their metabolites were determined by high-performance liquid chromatography (HPLC) as described previously [74,75,76], with slight modifications. Purchased standards for all detected compounds were used for quantification.

### 4.10. Statistical Analysis

Mean values were compared between WT and KO animals using two-tailed Student’s *t*-tests with Bonferroni correction for multiple comparisons, where appropriate. Detailed information on used statistical tests can be found in the corresponding figure captions. *N* always refers to the number of animals used in a certain experiment. Probability (*p*) values ≤ 0.05 were considered statistically significant.

## 5. Conclusions

Overall, our data reassert that cardiac defects in human ether-lipid-deficient patients are not a coincidence and verify that ether lipid deficiency causes a complex, multifaceted, and presumably multifactorial cardiac phenotype. Manifestations and cardiac abnormalities in human RCDP vary considerably from patient to patient. The murine phenotype partly overlaps with human pathology, like the impaired closing of the ventricular septum, but also includes features not observed in RCDP patients, like the prolonged QRS duration in the ECG. One reason for these discrepancies may be the fact that *Gnpat* KO mice cannot produce any ether lipids, whereas cardiac pathology in RCDP patients is usually evaluated in less severe cases with residual biosynthesis of ether lipids who survive infancy. Alternatively, differences between species might play a central role, as murine muscle tissue has been shown to contain a lower relative amount of plasmalogens than human muscle [77]. Nevertheless, our findings emphasize that cardiac dysfunction is a major aspect of the pathology of ether lipid deficiency that should be considered in the clinical evaluation of patients as well as in the development of therapeutic strategies.

## Figures and Tables

**Table 1:** Summary of parameters determined in functional investigations.

Parameter	Change in *Gnpat* KO Mice
Heart weight/body weight	↑
**MRI**	
LV end-diastolic volume	↓
LV end-systolic volume	=
RV end-diastolic volume	=
LV mass	↓
LV end-diastolic diameter	↓
LV end-systolic diameter	=
Ejection fraction	=
Stroke volume	↓
Wall thickness (systolic and diastolic)	=
Wall stress (diastolic)	↑
Wall stress (systolic)	=
**In vivo hemodynamic examination**	
LV systolic pressure	↑
LV end-diastolic pressure	↑
**Echocardiography**	
Ejection fraction	=
Fractional shortening	=
Stroke volume	↓
Wall thickness (systolic and diastolic)	=
**ECG**	
P wave duration	↑
QRS length	↑
R/S ratio	↓
Dispersion	↑
Wenckebach point	↑

↑, increased; ↓, decreased; =, not significantly changed. LV, left ventricle; RV, right ventricle.

**Table 2:** ECG characteristics of ventricular conduction in WT and *Gnpat* KO mice.

	WT	*Gnpat* KO
R/S ratio	10.61 ± 14.33 (*n* = 45)	4.96 ± 6.11 * (*n* = 40)
Dispersion (QRS)	3.17 ± 1.38 ms (*n* = 28)	4.88 ± 2.33 ms ** (*n* = 25)
Dispersion (QT1)	4.68 ± 2.04 ms (*n* = 36)	6.89 ± 4.26 ms * (*n* = 27)

All values are presented as means ± SD. Statistical analysis was performed using two-tailed Student’s *t*-tests followed by Bonferroni correction for the comparison of the different intervals in the case of dispersion. ** *p* < 0.01; * *p* < 0.05.

**Table 3:** Cardiac pathology in human ether lipid deficiency (RCDP).

Reference	Fraction of Patients with Cardiac Pathology/Total Patients	Main Pathological Findings
[14]	13/18	ASD, MVP, PDA, PS, ToF
[15]	9/14	ASD, MVP, ToF, VSD, ARD
[27]	5/11	ASD, MVP, PDA, PS
This study	1/2 *	ASD

* Refers to those patients who have not been reported before and for whom echocardiography reports were available. ARD, aortic root dilatation; ASD, atrial septal defect; MVP, mitral valve prolapse; PDA, patent ductus arteriosus; PPS, pulmonary stenosis; ToF, tetralogy of Fallot; VSD, ventricular septal defect.

**Table 4:** QTc intervals in RCDP type 1 patients.

RCDP Patient (Age in yrs)	QTc Interval (s)	Sinus Rhythm	Comment
1 (12)	0.337 *	Marked sinus arrhythmia	ST elevation (early repolarization normal variant)
2 (7)	0.400	Normal sinus rhythm	Normal ECG
3 (3)	0.315 *	Normal sinus rhythm	+T in V1 (RVH with pressure overload)
4 (3)	0.331 *	Normal sinus rhythm	RSr’ in V1 (mild intraventricular conduction delay—normal variant)
5 (3)	0.395	Normal sinus rhythm	rSR’ in V1 (RVH with volume overload ^1^ or mild conduction delay ^2^)
6 (31)	0.392	Normal sinus rhythm	Deep S in V5-V6 with no other signs of RVH (likely artifact of chest abnormality)
7 (21)	0.395	Normal sinus rhythm	rSR’ in V1 (RVH with volume overload ^1^ or mild conduction delay ^2^, borderline tall p wave in lead II—possible RA enlargement)
8 (5)	0.371	Sinus arrhythmia	Normal ECG
9 (10)	0.381	Normal sinus rhythm	Normal ECG
10 (3)	0.388	Normal sinus rhythm	rSR’ in V1 (RVH with volume overload or mild conduction delay)

RA, right atrium; RCDP, rhizomelic chondrodysplasia punctata; RVH, right ventricular hypertrophy; * Meets the criterion for short QTc interval (QTc ≤ 340 ms) [29]. ^1^ In this population, the most likely cause of RVH with volume overload is an atrial septal defect. ^2^ Note that rSr’ with mild conduction delay may not be clinically significant.

## Data Availability

The data presented in this study are available on request from the corresponding author.

## Supplementary Materials

The supporting information can be downloaded at: https://www.mdpi.com/article/10.3390/ijms24031884/s1.

## Abbreviations

AV, atrioventricular; Cx, connexin; ECG, electrocardiography; EDV, end-diastolic volume; ESV, end-systolic volume; FCS, fetal calf serum; GNPAT, glyceronephosphate O-acyltransferase; HW/BW, heart weight/body weight; KO, knockout; LV, left ventricle; LVM, left ventricle mass; MRI, magnetic resonance imaging; PB, Soerensen’s phosphate buffer; PCR, polymerase chain reaction; PFA, paraformaldehyde; PE, phosphatidylethanolamine; RCDP, rhizomelic chondrodysplasia punctata; WT, wild type.
