# Supplementary material for: Overlapping and Distinct Features of Cardiac Pathology in Inherited Human and Murine Ether Lipid Deficiency
Source: Int J Mol Sci. 2023 Jan 18;24(3):1884. doi: 10.3390/ijms24031884 (PMC9914995; doi:10.3390/ijms24031884)
Supplement: Supplementary file 1 [file ijms-24-01884-s001.zip › Supplementary Material Dorninger et al..pdf]

Supplementary Information

Overlapping and distinct features of cardiac pathology in inherited human and murine ether lipid deficiency

Fabian Dorninger, Attila Kiss, Peter Rothauer, Alexander Stiglbauer-Tscholakoff, Stefan Kummer, Wedad Fallatah, Mireia Perera-Gonzalez, Ouafa Hamza, Theresa König, Michael B. Bober, Tiscar Cavalle-Garrido, Nancy E. Braverman, Sonja Forss-Petter, Christian Pifl, Jan Bauer, Reginald E. Bittner, Thomas H. Helbich, Bruno K. Podesser, Hannes Todt, Johannes Berger

Supplementary Figure S1

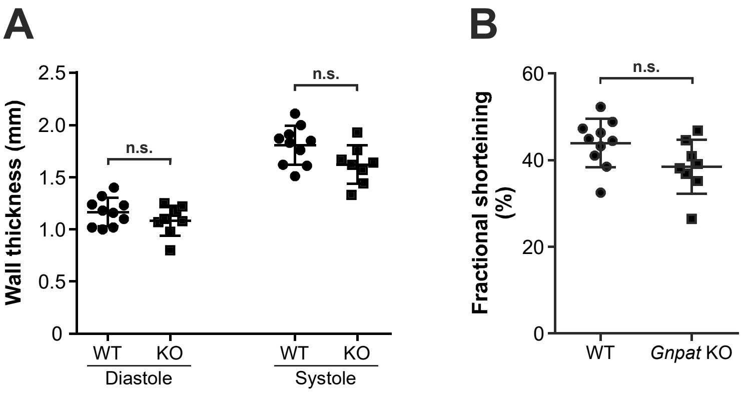

Supplementary Figure S1. *Echocardiographic analysis of WT and Gnpat KO mice.* (A) Myocardial diameter in middle-aged WT (*n*=12, circles) and *Gnpat* KO (*n*=10, squares) mice was determined during systole and diastole using the wall trace measurement tool of the echocardiography software. Each data point represents one animal, and group means ± SD are shown. Statistical analysis was performed using two-tailed Student's *t*-tests followed by Bonferroni-Holm correction for multiple comparisons. (B) Fractional shortening was calculated automatically in the same cohort of mice and statistical analysis was performed using a two-tailed Student's *t*-test. *n.s.*, not significant

Supplementary Figure S2

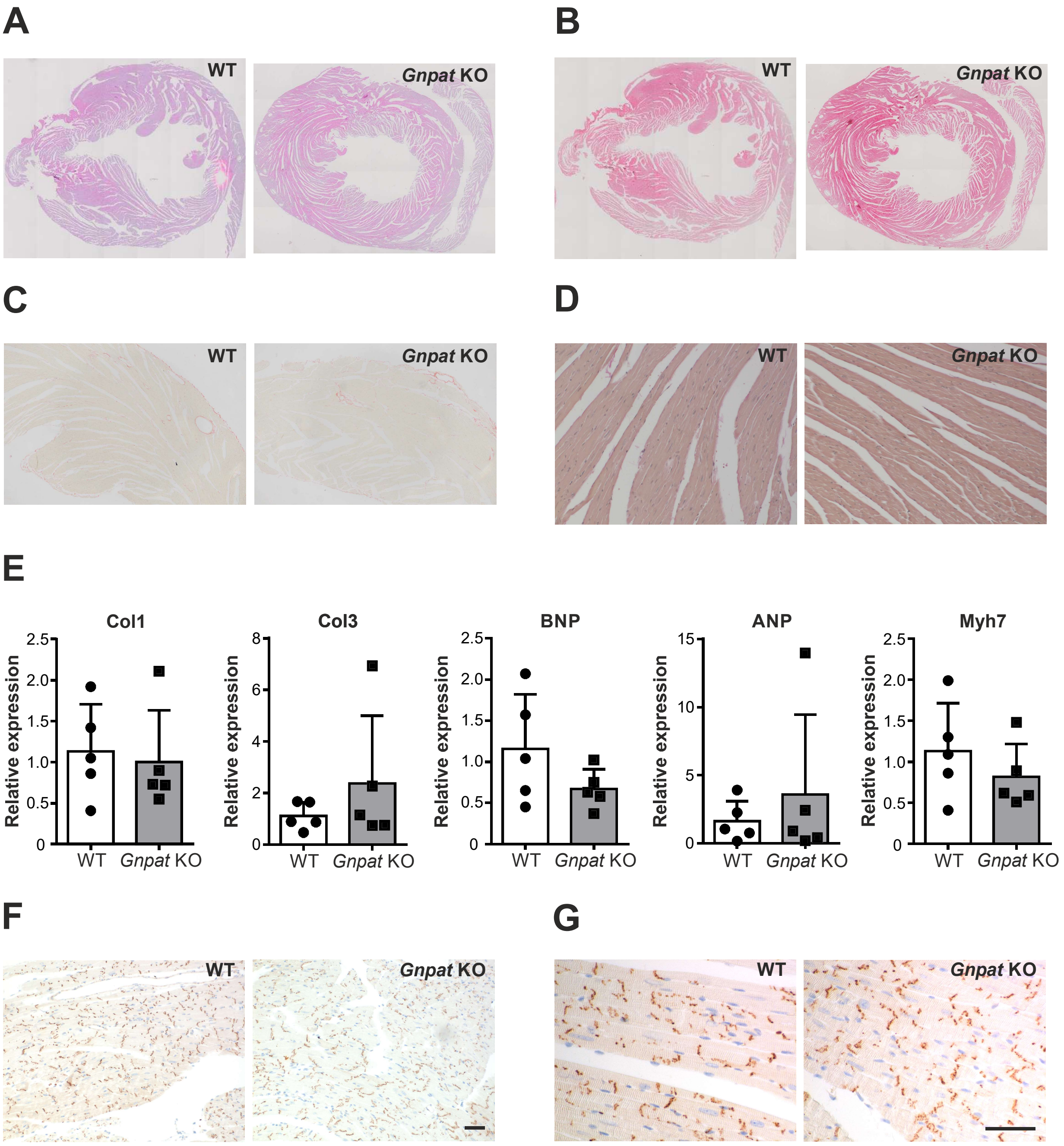

Supplementary Figure S2: *No marked fibrosis in ether lipid-deficient cardiac tissue.* (A-D) Representative images of paraffin-embedded tissue sections from WT and *Gnpat* KO mice (*n*=5/genotype, age 12-13 months) stained with H&E (A), Masson-Goldner trichrome stain (B), Sirius Red (C) and van Gieson's stain (D). (E) Expression of fibrosis markers was analyzed in cardiac tissue from aged WT and *Gnpat* KO mice (12-16.5 months) using quantitative real-time PCR to determine the mRNA levels. Relative values (to the housekeeping genes *β-actin* and *Gapdh*) were calculated using the 2−ΔΔCt method for collagen I (*Col I*), collagen III (*Col III*), B-type/brain natriuretic peptide (*BNP*), atrial natriuretic peptide (*Anp*) and myosin heavy chain 7 (*Myh7*), and results are shown as data for individual mice (*n*=5/genotype) together with group means ± SD. No statistically significant differences between the genotypes were found (two-tailed Student's *t*-tests). (F, G) Paraffin-embedded sections of cardiac tissue derived from WT and *Gnpat* KO mice (*n*=5/genotype, age 12-13 months) were immunohistochemically stained for Cx43 and representative light microscopy images are shown at 10x (F) and 25x magnification (G). Scale bar = 50 µm

Supplementary Figure S3

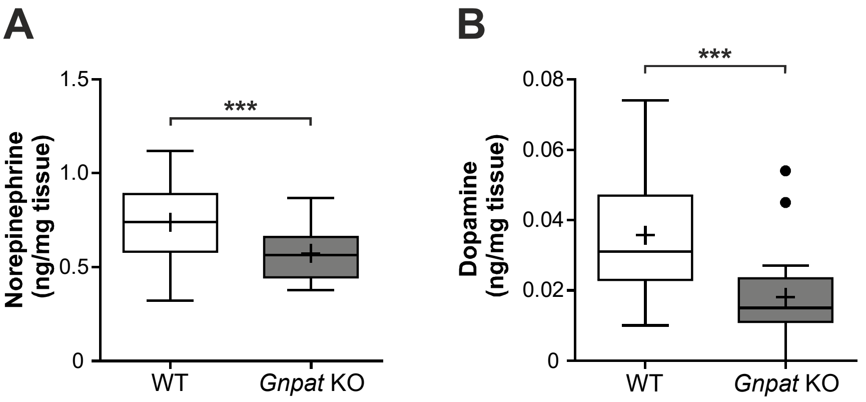

Supplementary Figure S3: *Depletion of monoamine neurotransmitters in ether lipid-deficient cardiac tissue*. The levels of norepinephrine (A) and dopamine (B) were determined in cardiac homogenates from aged WT (*n*=31) and *Gnpat* KO (*n*=25) mice using HPLC. The box plot is drawn according to Tukey's method, with median (horizontal line within the box) and mean (+) values indicated; statistical analysis was performed using a two-tailed Student's t-test. ***$P < 0.001$

Supplementary Figure S4

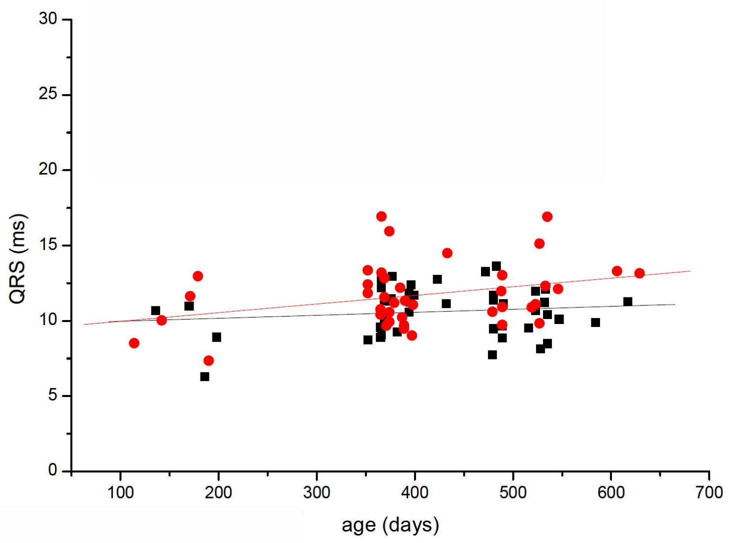

Supplementary Figure S4. *Correlation between age and QRS duration in ether lipid-deficient mice.* QRS duration (in ms) of all mice exposed to resting ECG examination is presented as a function of age (in days). Correlation analysis showed a statistically significant relationship between the two parameters (r = 0.32; *P* = 0.04) for the *Gnpat* KO group (red circles) but not the WT group (black squares).

Supplementary Table S1. *Summary of cardiac examinations (mainly echo reports) of patients with RCDP.* Data was extracted from the RhizoKids registry, maintained at the Skeletal Dysplasia Center, Nemours Children's Hospital, Wilmington, Delaware, USA. As indicated in the table, several patients have been reported previously but current follow-ups are presented here. Cardiac features are not listed at follow-up visits, if they were already mentioned at earlier visits and did not change with time.
